# Tularemia, a re-emerging infectious disease in Iran and neighboring countrie

**DOI:** 10.4178/epih/e2015011

**Published:** 2015-02-22

**Authors:** Afsaneh Zargar, Max Maurin, Ehsan Mostafavi

**Affiliations:** 1 Department of Epidemiology, Pasteur Institute of Iran, Tehran, Iran; 2 Research Centre for Emerging and Reemerging Infectious Diseases, Pasteur Institute of Iran, Akanlu, Kabudar-Ahang, Hamadan, Iran; 3 Centre National de Référence des Francisella, Laboratoire de Bactériologie, Département des Agents Infectieux, Institut de Biologie et Pathologie, Centre Hospitalier Universitaire de Grenoble, Université Joseph Fourier, Grenoble, France; 4 Laboratoire Adaptation et Pathogénie des Microorganismes, CNRS UMR 5163, Grenoble, France

**Keywords:** Tularemia, *Francisella tularensis*, Bacterial infections, Rodentia

## Abstract

**OBJECTIVES:**

Tularemia is a zoonotic disease transmitted by direct contact with infected animals and through arthropod bites, inhalation of contaminated aerosols, ingestion of contaminated meat or water, and skin contact with any infected material. It is widespread throughout the northern hemisphere, including Iran and its neighbors to the north, northeast, and northwest.

**METHODS:**

In this paper, the epidemiology of tularemia as a re-emerging infectious disease in the world with a focus on Iran and the neighboring countries is reviewed.

**RESULTS:**

In Iran, positive serological tests were first reported in 1973, in wildlife and domestic livestock in the northwestern and southeastern parts of the country. The first human case was reported in 1980 in the southwest of Iran, and recent studies conducted among at-risk populations in the western, southeastern, and southwestern parts of Iran revealed seroprevalences of 14.4, 6.52, and 6%, respectively.

**CONCLUSIONS:**

Several factors may explain the absence of reported tularemia cases in Iran since 1980. Tularemia may be underdiagnosed in Iran because *Francisella tularensis* subspecies *holarctica* is likely to be the major etiological agent and usually causes mild to moderately severe disease. Furthermore, tularemia is not a disease extensively studied in the medical educational system in Iran, and empirical therapy may be effective in many cases. Finally, it should be noted that laboratories capable of diagnosing tularemia have only been established in the last few years. Since both recent and older studies have consistently found tularemia antibodies in humans and animals, the surveillance of this disease should receive more attention. In particular, it would be worthwhile for clinical researchers to confirm tularemia cases more often by isolating *F. tularensis* from infected humans and animals.

## INTRODUCTION

Tularemia is a zoonotic disease caused by *Francisella tularensis*, a gram-negative, intracellular bacterium [1] transmitted through direct contact with infected wildlife or domestic animals, arthropod bites (mostly ticks, but also flies and mosquitoes), inhalation of contaminated aerosols, ingestion of contaminated meat or water, and touching infected material. Direct human-to-human transmission has never been reported [2]. Tularemia has been isolated from 200 species of mammals, birds, reptiles, and fishes [2].

Laboratory workers, farmers, veterinarians, hunters, foresters, cooks, and butchers are at risk of tularemia infection [3]. *F. tularensis* is widespread in soil and water environments, where it can survive for up to 140 days [4]. However, no agreement exists about whether water can be considered a reservoir for this bacterium [5,6]. Since *F. tularensis* is highly virulent and can be spread via aerosols, causing severe pneumonia, it is a potential biological weapon [1].

The aim of this paper is to review the epidemiology of tularemia as a re-emerging infectious disease, with a focus on Iran and its neighboring countries.

### Clinical manifestations of tularemia

The infectious dose of *F. tularensis* in humans is 25 bacteria for aerosol transmission and 100 bacteria for oral transmission [7,8]. After infection, the latent phase depends on the virulence and number of bacteria that entered the body, but most often lasts between three and six days [1]. The clinical manifestations of tularemia depend on the route through which the bacterium entered the body and are usually ulceroglandular or typhoidal, although oculoglandular, oropharyngeal, and pneumonic forms have also been reported [2]. The ulceroglandular form, which represents 75 to 85% of all tularemia cases, corresponds to a regional lymphadenopathy with a painful maculopapular lesion that evolves to an eschar at sites of skin infection; it can also occasionally develop into systemic disease with a 5 to 15% case fatality rate. The typhoidal form, which is an acute form that occurs in 5 to 15% of all cases of tularemia, is a febrile systemic disease that can include gastrointestinal and respiratory symptoms. It is associated with a case fatality rate of approximately 35%. Early diagnosis of tularemia can help prevent fatal outcomes [9].

### *Francisella tularensis* subspecies

*F. tularensis* currently includes four subspecies ([subsp.] *tularensis*, *holarctica*, *mediasiatica*, and *novicida*) with different phenotypic traits, virulence, and geographical distributions [7]. Only two subsp. (*tularensis* [type A] and *holarctica* [type B]) are causative agents of human tularemia.

Type A strains are highly virulent in humans. They are predominantly found in North America, where mice, rats, hares, and squirrels are natural reservoirs of these bacteria [10,11]. The case fatality rate of type A infections is 5 to 15% without antibiotic therapy (30 to 60% in cases involving severe pneumonia or septicemia), and <2% with antibiotic therapy. Type A strains have been separated into genotypes (AI and AII) and subtypes (e.g., AIa and AIb). In the US, type AI predominates in the central and eastern parts of the country, whereas type AII predominates in the western part [3,12]. The subtype AIb is currently considered the most virulent.

Type B strains are usually associated with less severe forms of tularemia, with a case fatality rate <1% in Europe and Asia. However, a 7% fatality rate was recently reported for type B infections in the US [13,14]. Tularemia is mostly transmitted through contact with contaminated dead or alive animals, arthropod bites (mainly ticks and mosquitoes), ingestion of contaminated water or food, and, rarely, inhalation of contaminated aerosols. Mosquito bites are considered the main route of transmission in Russia and Scandinavia [15,16]. Mosquitoes can be infected with *F. tularensis* during their life as larvae in stagnant water, and then transmit the disease as adults [11].

*F. tularensis* subsp. *novicida* is not considered an etiological agent of tularemia. However, it has been occasionally associated with severe systemic disease in immunosuppressed patients [17]. It has been isolated from contaminated water, mainly in the US, and more rarely in Europe, Japan, China, and Australia [1,17,18].

*F. tularensis* subsp. *mediasiatica* has only been isolated in Kazakhstan and Turkmenistan. Only a few studies have been conducted on this subsp. [19], but experiments in hares have shown that it is less virulent than subsp. *holarctica* (type B) [20].

### The geographic distribution of tularemia worldwide

**Figure 1.**
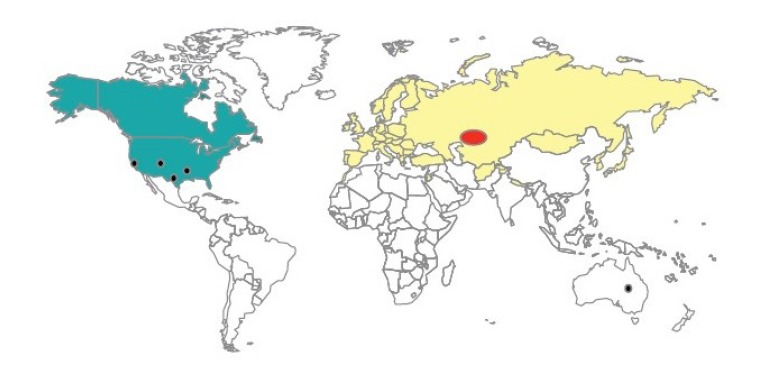
The global distribution of disease caused by *Francisella tularensis* subspecies. The different shadings represent the distributions of both type A and type B (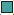), type B (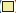) novicida (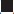), and mediasiatica (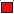). Source from Petersen JM, et al. Vet Res 2005;36:455-467 [23].

Tularemia is widespread in the northern hemisphere, mostly occurring in forests and mountainous regions [21]. The main endemic foci of the disease are Russia, Kazakhstan, Turkmenistan, Finland, and Sweden [22,23], and annual reports of tularemia incidence are published in the eastern European countries. Tularemia is considered a rare disease in western European countries, but recent outbreaks have occurred in Spain, involving hundreds of cases. The disease is also prevalent in Japan and in the northwest and northeast of China. Tularemia cases have been systematically reported in recent decades in Sweden, the US, and Canada (Figure 1) [24-27]. An outbreak of tularemia also occurred in South Korea in 1998 [28].

During an outbreak occurring in 1966 in Sweden, more than 600 tularemia cases were reported after the inhalation of contaminated aerosols on a farm [29]. In 2000 and 2003, following the war in Kosovo, outbreaks were seen in Kosovo in areas under the control of the United Nations, with 300 reported cases. The areas in question were not previously known as areas where tularemia was endemic [30].

Between 1997 and 2008, outbreaks were reported in America, Spain, Yugoslavia, Russia, Kosovo, Turkey, Switzerland, Ukraine, France, Canada, Bulgaria, Germany, Norway, Czech, Finland, Japan, and Slovakia [31].

### The status of tularemia in countries neighboring Iran

In addition to the countries that were formerly part of the Soviet Union, tularemia has been reported in Asia in Turkey, China, Japan, and Iran [32].

Tularemia has been reported to the west of Iran (Turkey), as well as in countries bordering Iran to the north (Turkmenistan, Kazakhstan, and Azerbaijan) and to the east (Afghanistan).

Of the countries neighboring Iran, the greatest prevalence of tularemia has been reported in Turkey, where *F. tularensis* subsp. *holarctica* has been isolated [33]. Four major outbreaks were reported between 1936 and 1953 in the western, southwestern, southeastern, and northwestern parts of Turkey [34]. After several years during which no cases were reported, tularemia was again reported in 1988 in multiple regions of Turkey [35,36]. Between 1988 and 2009, 1,300 cases of tularemia have been reported from various regions of Turkey [8,34].

Almost all cases of tularemia in Turkey have been oropharyngeal (Table 1); however, ulceroglandular and ocular forms of tularemia have also been diagnosed in Bursa, in the northwest of Turkey [34]. The main transmission route in Turkey is through drinking contaminated water. It has been hypothesized that water becomes contaminated by secretions from rodents and hares [39-41]. The potential role of migrating birds in the transmission of the disease may be explained through the transfer of infected ticks or the direct contamination of water [42].

Although outbreaks have been reported in the northern and eastern regions of Turkey, most cases in Turkey have occurred in its northwestern and central regions. Both sexes and all age groups have been affected by the disease, and most cases have been reported in the autumn and winter [35,36].

Tularemia has been reported once in Afghanistan, in a porcupine in 1973 [43]. Tick bites, rodents, sheep, and water transmission routes have been responsible for human tularemia cases in Armenia [44].

Tularemia has been carefully studied In Azerbaijan, where *F. tularensis* has been isolated from wild mammals, ticks, fleas, and water [45]. A study in Azerbaijan reported a tularemia seroprevalence of 15.5% [45].

In addition, *F. tularensis* subsp. *mediasiatica* has occasionally been isolated in central Asia (Turkmenistan and Kazakhstan) [46]. This subsp. is found in regions around the Amu Darya in Turkmenistan, and it is believed that it can be found in southern areas of central Asia as well [32,45].

### The status of tularemia in Iran (Figure 2)

In a national study conducted in 1973 in Iran, samples from more than 4,600 wild mammals and 200 sheep were collected from 47 regions. Attempts were made to isolate *F. tularensis* from the spleens of 3,548 animals, but no cultures were positive. Tularemia antibodies were found in eight sheep and three cows in the northwest of Iran, and in one porcupine in the southeast of Iran [44].

The first human case of tularemia (a glandular form) was reported in 1980 in Marivan, in the southwestern part of Kurdistan Province. The case was a soldier stationed in that area for his military service, who suffered from symptoms including fatigue, myalgia, headache, anorexia, and inguinal lymphadenopathy [47].

In a study in Kurdistan Province, in the west of Iran, a study was carried out in 2011 and 2012 analyzing 100 patients who were referred to laboratories, 50 hunters and their families, 50 butchers and slaughterhouse workers, and 50 persons working in public healthcare centers in Sarvabad, Marivan, and Sanandaj. It was found that 42% of the subjects kept livestock. Tularemia antibodies were found in 14.4% of the 250 samples that were analyzed. The highest seroprevalence was found among hunters (18%), whereas the lowest was observed among persons working in healthcare centers (12%). The prevalence of tularemia among people exposed to foxes (either hunters or persons consuming fox meat) was significantly higher than among people with no exposure to foxes (25 vs. 8.65%) [48].

A study in 2011 that sampled 10 urban areas in Sistan and Baluchestan Province revealed tularemia seroprevalences of 5 and 9.38% among 184 butchers and slaughterhouse workers, respectively [49].

The same year, a study was conducted in Chaharmahal-Bakhtiari in 183 children between two and 18 years of age. This study revealed a seroprevalence of 6% for IgG antibodies against tularemia. However, none of the children presented clinical symptoms such as fever, ulcer, or lymphadenopathy. The subjects in this study lived in rural areas. Their parents worked as farmers or shepherds and they reported having close contact with animals such as dogs, cows, sheep, or other domestic animals [50].

## CONCLUSION

According to studies recently conducted in Iran, the tularemia seroprevalence in at-risk populations has been found to be 14.4% [48], 6.52% [49], and 6% [50] in the west, southeast, and southwest of Iran, respectively. This may indicate that the tularemia seroprevalence is higher in Iran than in many other countries where tularemia is endemic; for instance, a tularemia seroprevalence of 2% was reported among hunters in Germany [51] and among trappers in Canada [52], a seroprevalence of 1 to 7% was reported among hunters and rural inhabitants in Turkey [53,54], and a seroprevalence of 9% was reported in landscapers in the US [55]. These findings are in sharp contrast with the fact that no tularemia cases have been reported in Iran since 1980.

No reservoir of *F. tularensis* has ever been precisely identified in Iran. However, in each country that has been studied, the prevalence of the disease strongly depends on the different traditions and customs of the inhabitants [56,57]. Multiple studies in Turkey have revealed that outbreaks in Turkey generally originate from water sources. Migrating birds may play an important role in outbreaks caused by water contamination, because birds may contaminate water with their feces [39,40]. A serological study in the west of Iran showed that the seroprevalence of tularemia was highest among local hunters (18%). This study also showed a significant relationship between seroprevalence and eating meat from wild animals, such as foxes [48].

Type B tularemia is usually a benign disease [45,55], and *F. tularensis* subsp. *holarctica* is likely the etiological agent of tularemia in Iran, although no strain has yet been isolated in Iran. The clinical manifestations of tularemia in Iran are thus probably poorly specific and of low severity. Empirical therapies have probably been administrated in patients with suspected tularemia, especially because laboratories capable of diagnosing tularemia have only been established in the past few years. All these factors may explain the under-diagnosis of tularemia in Iran in recent decades [48].

Since annual reports of tularemia cases are published in countries that border Iran to the north, such as Turkey, more careful attention should be paid to the surveillance of tularemia in Iran, particularly in rural regions. Tularemia should receive a greater emphasis in the medical educational system of Iran. It is highly recommended that physicians and healthcare workers become more knowledgeable about the natural cycle of *F. tularensis* and the clinical manifestations of tularemia, in order to help them recognize the disease. Improvements in the Iranian healthcare system could lead to more reports of tularemia. Moreover, further studies are needed to better clarify the epidemiology of tularemia in Iran. The tularemia seroprevalence in other parts of Iran should be assessed, and the bacterium should be isolated from sources such as water, domestic livestock, and wild animals, in order to characterize the common subsp. and natural reservoirs of *F. tularensis* in Iran.

## Figures and Tables

**Figure 2.**
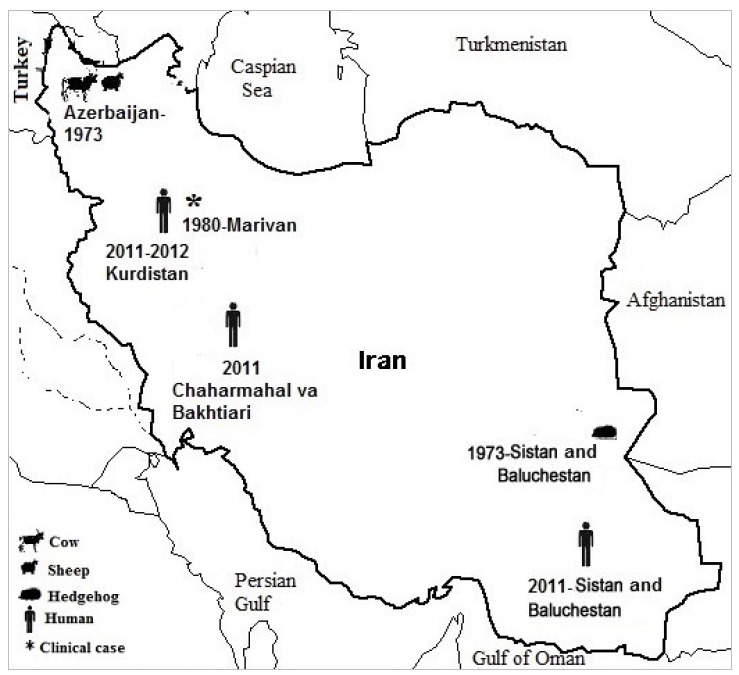
Distribution of clinical or serological positive cases of tularemia reported in different parts of Iran.

**Table 1.** Characteristics of tularemia outbreaks reported in Turkey

Region	Geographical location in Turkey	Transmission	Clinical form	No. of cases	Year
Kirklareli-Tekirdağ	Northwest	Water	Oropharyngeal	133	1936
Bingöl-Tatvan-Reşadiye	East-Southeast-Northeast	Food	Oropharyngeal	6	1937
Kırklareli-Lüleburgaz	Northwest	Water	Oropharyngeal	15	1945
Antalya-Bademagaci	Southwest	Water	Oropharyngeal	200	1953
Bursa	Northwest	Water	Oropharyngeal	205	1988-1998
Ankara-Ayaş-Yağmurdede	Central	Water	Oropharyngeal	16	1997
Düzce-Akçakoca	Northwest	Water	Ulceroglandular, oropharyngeal	33	2000, 2005
Bolu-Gerede-Yazikara	Northwest	Water	Oropharyngeal	21	2001
Amasya-Suluova	North	Unknown	Oropharyngeal, glandular	86	2004
Zonguldak Kastamonu-Bartin	Northwest	Water	Oropharyngeal	122	2004-2005
Kars-Sarıkamış	East	Water	Oropharyngeal	56	2004-2005
Kocaeli-Gölcük	Northwest	Water	Oropharyngeal	145	2004-2005
Kocaeli-Karamürsel-Pazarköy	Northwest	Water	Oropharyngeal	17	2005
Edirne-Lalapaşa-Demirköy	Northwest	Water	Oropharyngeal	10	2005
Samsun-Havza	North	Water	Oropharyngeal, glandular	75	2005-2007
Sakarya-Kocadöngel	Northwest	Water	Oropharyngeal	63	2005-2006
Bolu-Gerede-Nuhören	Northwest	Water	Unknown	6	2006
Tokat	North	Water	Oropharyngeal, ulceroglandular	23	2005, 2010
Sivas	Central	Water	Oropharyngeal	29	2008-2010
Çanakkale	Northwest	Water	Oropharyngeal	36	2009
Çankırı-Çerkeş-Kadıözü	Central	Water	Oropharyngeal	18	2009
Tekirdağ-Hayrabolu-Muzruplu	Northwest	Water	Oropharyngeal	8	2010
Konya	Central	Water	Oropharyngeal	40	2009-2010
Central Anatolia	Central	Water	Oropharyngeal, glandular, oculoglandular	139	2009-2011
Total				1,441	1936-2011

Source from Akalın H, et al. Int J Infect Dis 2009;13:547-551 [37]; Gürcan S. Balkan Med J 2014;31:3-10 [38].
